# Supplementary material for: Targeted Delivery Prodigiosin to Choriocarcinoma by Peptide-Guided Dendrigraft Poly-l-lysines Nanoparticles
Source: Int J Mol Sci. 2019 Nov 1;20(21):5458. doi: 10.3390/ijms20215458 (PMC6862698; doi:10.3390/ijms20215458)
Supplement: Supplementary file 1 [file ijms-20-05458-s001.pdf]

Targeted Delivery Prodigiosin to Choriocarcinoma by Peptide-Guided Dendrigraft Poly-l-lysines Nanoparticles

Kai Zhao [1, 2, *], Dan Li [1, 2, 3, 4], Guogang Cheng [2, 3], Baozhen Zhang [3], Jinyu Han [2, 3, 5], Jie Chen [3], Baobei Wang [3], Mengxia Li [3], Tianxia Xiao [3], Jian Zhang [3], Dongpo Zhou [1, 2], Zheng jin [5], Xiujun Fan [3, *]

[1] *Engineering Research Center of Agricultural Microbiology Technology, Ministry of Education, Heilongjiang University, Harbin 150080, China*

[2] *Key Laboratory of Microbiology, College of Heilongjiang Province, School of Life Science, Heilongjiang University, Harbin 150080, China*

[3] *Guangdong Key Laboratory of Nanomedicine, Center for Reproduction and Health Development, Institute of Biomedicine and Biotechnology, Shenzhen Institutes of Advanced Technology, Chinese Academy of Sciences, Shenzhen 518052, China*

[4] *School of Pharmaceutical Sciences, Key Laboratory of Bioorganic Phosphorus Chemistry & Chemical Biology (Ministry of Education), Beijing Advanced Innovation Center for Structural Biology, Tsinghua University, Beijing 100084, China*

[5] *Key Laboratory of Chemical Engineering Process and Technology for High-efficiency Conversion, College of Chemistry and Material Sciences, Heilongjiang University, Harbin 150080, China*

Running title: Targeted delivered with plCSA-BP guided DGL

* Correspondence: zybin395@126.com (K.Z.); xj.fan@telomerescitech.com (X.F.); Tel.:

+86-451-86608586 (K.Z.); +86-755-86392360 (X.F.)

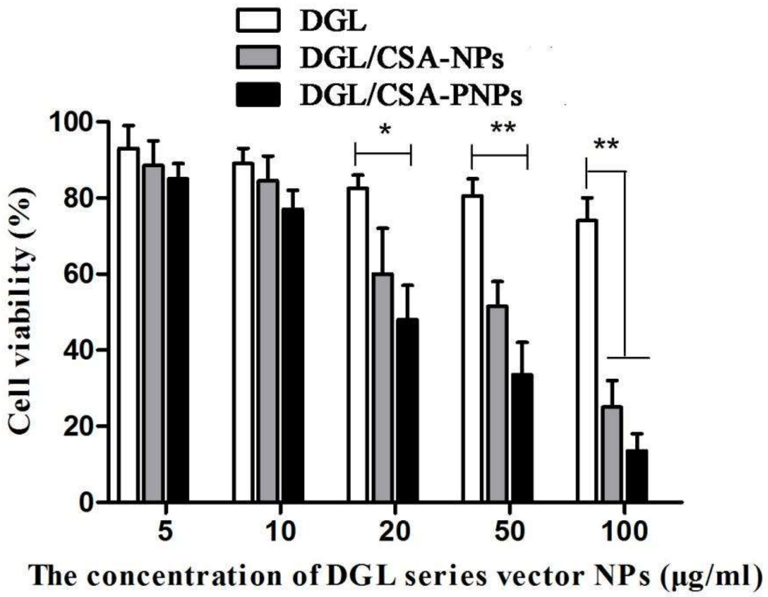

Figure S1. *In vitro* cell cytotoxicity evaluation of the DGL, DGL/CSA-NPs and DGL/CSA-PNPs. The cell viability measured by CCK-8 Kit performed 3 h in 293T cells after treated with DGL, DGL/CSA-NPs and DGL/CSA-PNPs at different concentrations. Data are presented as the mean ± standard deviation (n=4). "*" and "**" indicate statistically significant difference at P<0.05 and P<0.01 (Student's t test), respectively.

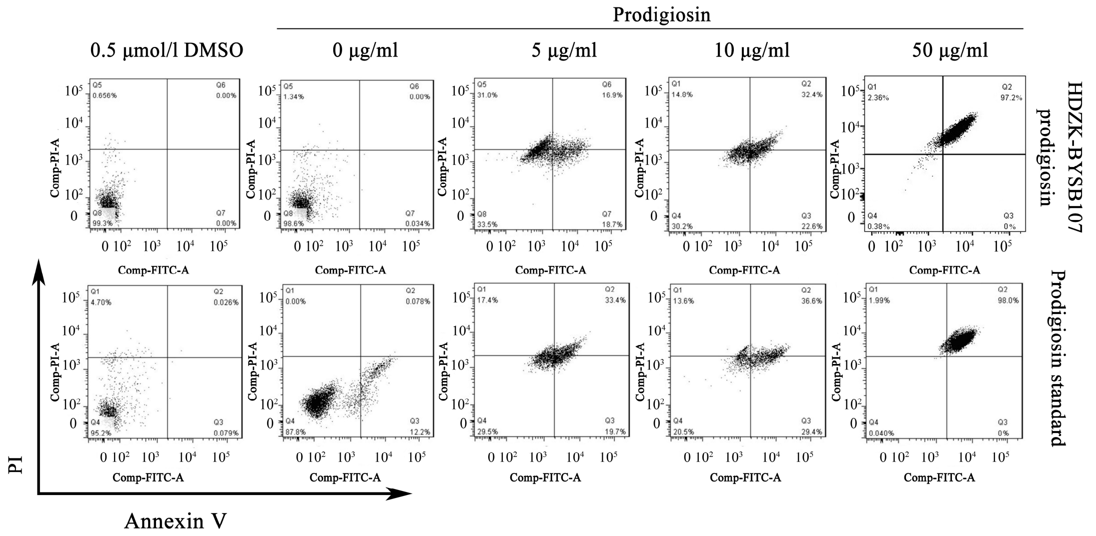

Figure S2. Prodigiosin from strain HDZK-BYSB107 and prodigiosin standard induced annexin V-based apoptosis in JEG3 cells *in vitro*. Representative flow cytometry-based dosed dose response of apoptosis profiles of annexin V/PI staining for JEG3 cells. Cells were treated with 0.5 μmol/l DMSO, 0, 5, 10, and 50 μg/ml prodigiosin for 24 h.

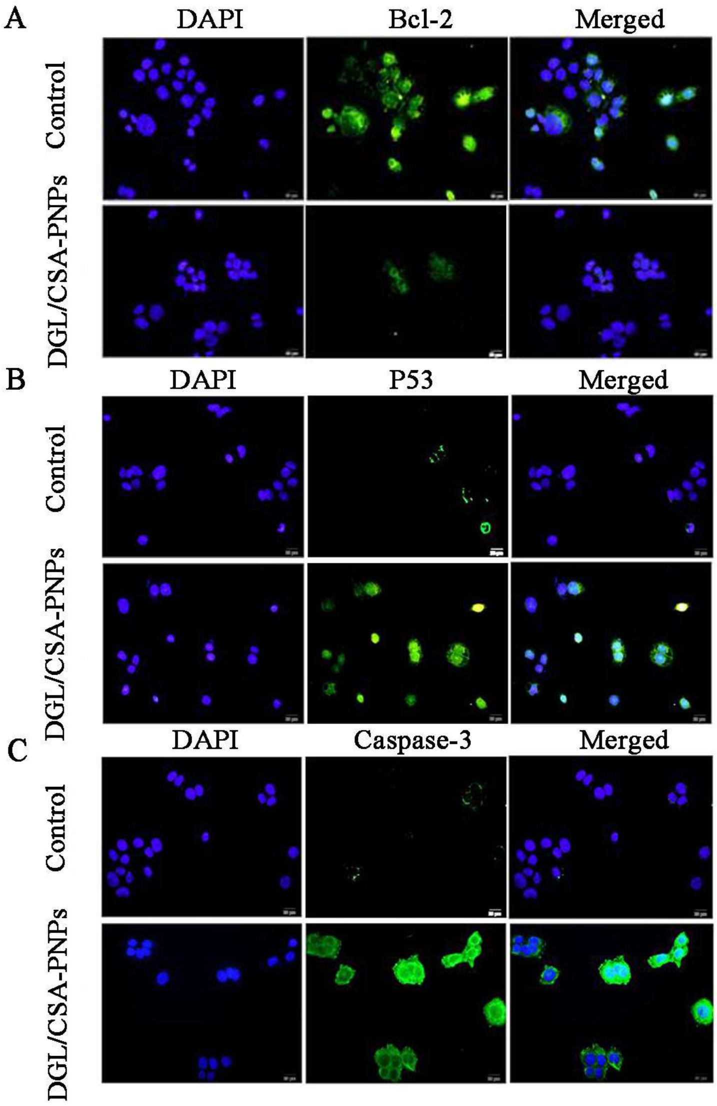

Figure S3. Immunofluorescence assay of bcl-2, P53 and caspase-3 induced by the DGL/CSA-PNPs in JEG3 cells. The DGL/CSA-PNPs induces up regulation of P53 (B), caspase-3 (C) and down regulation of bcl-2 (A) in JEG3 cells. Scale bar=50 μm.

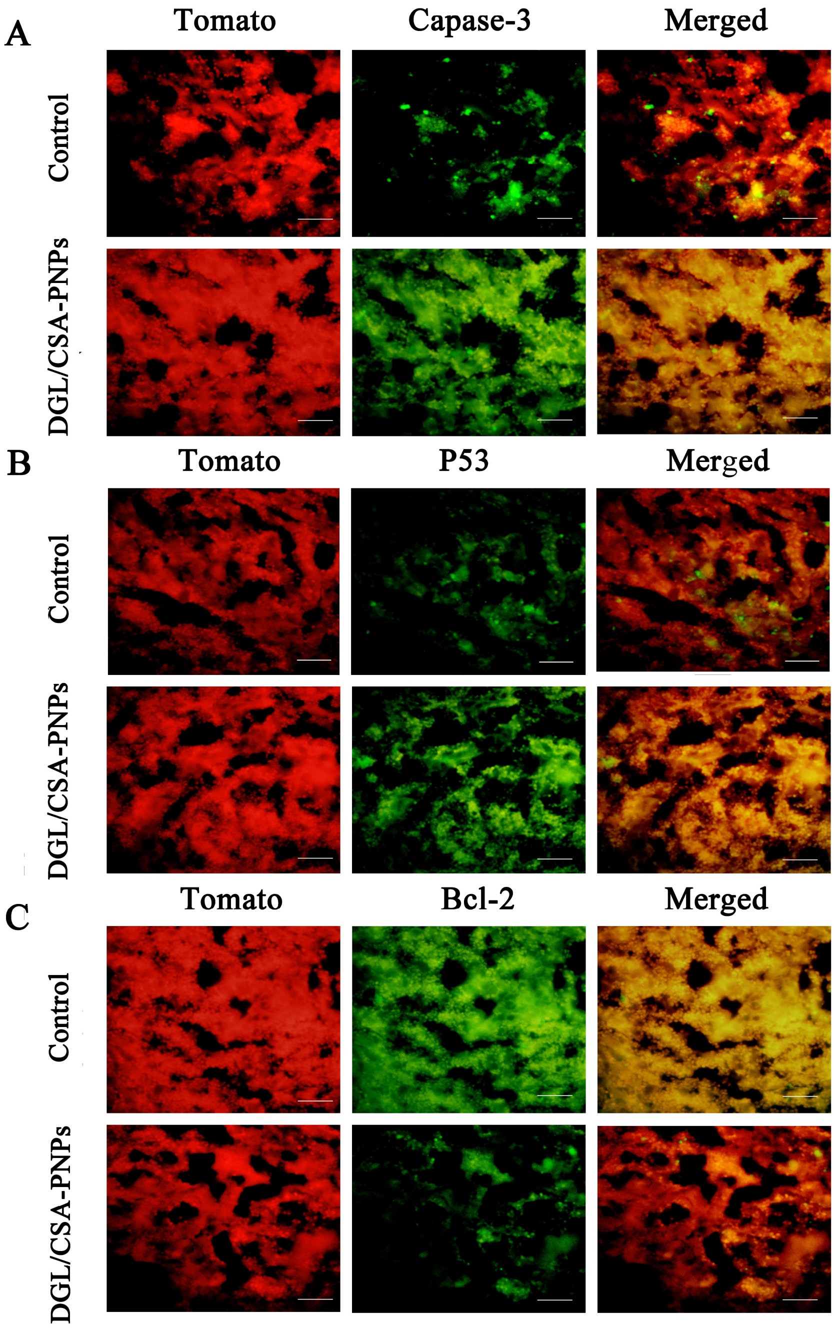

Figure S4. Immunofluorescence colocalization analysis of caspase-3, P53 and bcl-2 apoptosis pathway that induced by DGL/CSA-PNPs in JEG3 tumor that treated with DGL/CSA-PNPs and control group. DGL/CSA-PNPs induce up regulation of caspase-3 (A), P53 (B) and down regulation of bcl-2 (C) in JEG3 tumor. Scale bar=20 μm.
